# Statistical media optimization and cellulase production from marine *Bacillus* VITRKHB

**DOI:** 10.1007/s13205-013-0173-x

**Published:** 2014-01-03

**Authors:** Kunal Singh, Kumari Richa, Himadri Bose, Loganathan Karthik, Gaurav Kumar, Kokati Venkata Bhaskara Rao

**Affiliations:** Molecular and Microbiology Research Laboratory, Environmental Biotechnology Division, School of Bio Sciences and Technology, VIT University, Vellore, 632014 Tamil Nadu India

**Keywords:** *Bacillus* VITRKHB, Cellulase, Xylose, Yeast extract, RSM

## Abstract

Marine *Bacillus* species are potent producers of novel enzymes. Marine *Bacillus* VITRKHB was observed to be efficient for cellulolytic activity. It was employed for the production of extracellular cellulase. Cellulase was partially purified to 1.6-fold in a stepwise manner by ammonium sulfate precipitation, dialysis, and DEAE ion exchange chromatography. The molecular weight of purified protein was found to be about 33 kDa by SDS-PAGE. Its specific activity was recorded as 1.92 IU/mg. The effect of various carbon and nitrogen sources on cellulase production was investigated. The maximum enzyme activity was recorded in the fermentation media containing xylose as carbon source and beef extract as nitrogen source. The combined interactive effect of different variables on cellulase production was studied by response surface methodology. The optimized combination of variables for maximum enzyme activity was determined as; xylose 5.0 %, beef extract 6.9 %, pH 7.83, NaCl 1.17, and temperature 25.84 °C, after 24 h of incubation.

## Introduction

Cellulases are a class of enzymes primarily responsible for hydrolysis of cellulose into β-glucose. They are commonly employed in paper and pulp industry (Buchert et al. 1996), animal feed, and textile and detergent industry (Aygan and Arikan 2008; Sukumaran et al. 2005). They are also applied in the production of bioethanol (Wang et al. 2009) from lignocellulosic materials. The present enzyme toolbox is not sufficient to meet their industrial demand (Chakraborty et al. 2009). For this reason, exploring new and potent sources of cellulase is desirable. There are various sources of enzymes, although microorganisms are targeted nowadays due to their broad biochemical diversity, feasibility of mass culture, and ease of genetic manipulation (Eichler 2001) Microbial enzymes are believed to possess a higher degree of stability than those derived from plants and animals. Extreme conditions, viz, temperature and pH used during some of the industrial processes can degrade the enzyme or reduce its stability. Therefore, new techniques and process methodologies are implicated for isolation of enzymes exhibiting tolerance to such extreme conditions. This has propelled researchers to target extremophiles for enzymes carrying novel properties (Herbert 1992; Gupta and Roy 2002).

Marine ecosystem is a habitat with remarkably high and diverse microbial cell densities. Consequently, the microbial inhabitants of such environment must have adapted their cellular machinery to thrive in the extreme conditions of temperature, pressure, pH, salinity, etc. Earlier reports suggest their salt-tolerating capacity of above 1.7 M (Marhuenda-Egea and Bonete 2002), hyperthermostability (80–108 °C), barophilicity (60 MPa), cold adaptivity, and alkali stability. With increase in demand of the industrially valuable enzymes, along with selection of their potent source, their economical production has become a must. Improvement in fermentation conditions for maximizing cell density and overproduction of metabolite is frequently endeavored (Mukhopadhyay et al. 2008). But, this is a time-consuming process and does not explain the combined interactive effect of parameters involved with the fermentation process. To overcome these limitations, different initiatives have been undertaken. Among these initiatives, optimization of the process parameters affecting fermentation by statistically designed experimental strategies is an easy and rapid process. One such method is response surface methodology (RSM). It is a collection of experiments, mathematical methods, and statistical inference that evaluates the combined effect of all the factors participating in the fermentation process (Liu and Tzeng 1998; Elibol 2003). 3D plots for response surface assist visualization of the parameter interaction in a better manner (Zambare 2011). Consequently, optimal variables can be determined that give rise to desirable results. There are several reports on RSM-mediated optimization of enzyme production from microorganisms (Liu and Tzeng 1998; Elibol 2003; Zambare 2011). Hence, in the present study we describe response surface methodology-mediated optimization of the variables for an economical production of cellulase from marine eubacterial isolate inhabiting saltpan soil of the coast of Bay of Bengal near Andhra Pradesh.

## Materials and methods

### Chemicals

Media and chemicals were purchased from Hi Media chemicals, Mumbai, India; Merck Specialities Private Limited and Sisco Research Private Limited, Mumbai, India, respectively.

### Revival of strain *Bacillus* VITRKHB (JF960957)

Microbial isolate (*Bacillus* VITRKHB) was previously observed to be potent for some industrially desirable enzymes (Richa et al. 2013). It was subjected to further evaluation of its cellulolytic activity. It was preserved in glycerol stock kept at −20 °C. The culture was revived on nutrient agar media incubated at 37 °C for 24 h.

### Screening of the strain for cellulolytic activity

The *Bacillus* VITRKHB was screened for cellulolytic activity by growing it on a carboxy methyl cellulose (CMC) agar plate for 24 h at 37 °C. After incubation, 1 % iodine was poured on the starch agar plates and a combination of 0.1 ml HCl + 5 ml of 1 % iodine in 2 % KI was added to CMC agar plates. A clear zone of hydrolysis around the colony indicates a positive result.

### Production of cellulase

Enzyme production was carried out in the production media containing (g/l) CMC (20), peptone (20), NaCl (1), CaCl_2_ (0.005), MgSO_4_ (0.82), K_2_HPO_4_ (1.25), KH_2_PO_4_ (3), FeSO_4_ (0.01), ZnSO_4_ (0.005), MnCl_2_ (0.0001), and NH_4_Cl (1) (Wei et al. 2011). 10 ml of bacterial inoculum was added into 500 ml production medium and the flask was kept in a rotary shaker incubator at room temperature for 24 h. After incubation, the fermented broth was centrifuged at 10,000 rpm for 10 min in a cooling centrifuge. The supernatant was collected and subsequently used for the estimation of cellulase.

### Effect of different carbon sources on cellulase production and activity

The effect of five different carbon sources, viz., sucrose, fructose, xylose, starch, and lactose, on the production of enzymes was studied (Ashwini et al. 2011).

### Effect of different nitrogen sources on cellulase production and activity

The effect of five different nitrogen sources, viz., beef extract, yeast extract, nutrient broth, urea, and casein, on the production of enzymes was studied (Ashwini et al. 2011).

### Optimization by response surface methodology (RSM)

In the present study, statistically designed experiments were executed to optimize the process parameters influencing cellulase production. The five independent variables chosen for optimization were xylose (A), beef extract (B), pH (C), NaCl (D), and temperature (E). The total number of experiments suggested by the model was 31. The range of medium components is shown in Table 1. Each variable was set at two levels (−1 and +1). Trace element solution was constantly maintained (Wei et al. 2011). The experimental design was developed using Design Expert, version 7.0.7.1.

**Table 1:** Range of media components for RSM

	Low (−1)	High (+1)
(A) Xylose (g)	3	7
(B) Beef extract (g)	3	7
(C) pH	5	8
(D) NaCl (%)	1	4
(E) Temperature (°C)	20	35

### Protein estimation

The concentration of protein present in the fermentation media was investigated by Lowry’s method (Lowery et al. 1951).

### Enzyme assay

The enzyme activity of cellulase was studied according to the Denison and Koehn’s method (1977). One ml of 1 % CMC was added to 1 ml of cellulase and the tubes were incubated at 55 °C for 15 min in a water bath. Two ml of dinitrosalicyclic acid (DNS) was added so as to stop the reaction and the tubes were subsequently kept in a boiling water bath for 5 min. Thereafter, 1 ml of sodium potassium tartrate was added. Absorbance was recorded at 540 nm and after cooling the tubes.

### Partial purification

The RSM-optimized medium was further used for enzyme production. The crude extract was partially purified by ammonium sulfate precipitation. 100 ml of this extract was subjected to precipitation and brought to 80 % saturation by the slow addition of ammonium sulfate and gentle stirring at 4 °C. The precipitate was collected by centrifugation (10,000 rpm at 4 °C) and dissolved in 0.1 M Tris–HCl buffer (pH 7.83). The precipitate was desalted by dialysis. The solution was placed in a bag with a selectively permeable membrane (Dialysis membrane-150, Himedia laboratories Ltd; Mumbai, India) and immersed in a large volume of 0.1 M Tris–HCl buffer (pH 7.83) in stirring condition at 4 °C for 24 h.

The enzyme solution procured after dialysis was further purified by ion exchange chromatography. The solution was loaded on a DEAE cellulose column that was pre-equilibrated with 10 mM Tris–HCl buffer (pH 7.83). Proteins were eluted with 10 mM Tris–HCl and 0–2.0 M NaCl gradient. The eluted fractions (3 ml) were collected and absorbance was measured at 280 nm. Enzyme activity and concentration were estimated after each purification step. Furthermore, specific activity, yield, and fold purification was also calculated.

### SDS-PAGE

Sodium dodecyl sulfate polyacrylamide gel electrophoresis (SDS-PAGE) was performed for the purified protein to determine the molecular weight according to the method of Laemmli (1970) using a 10 % cross-linked polyacrylamide gel. The molecular weight was determined using Sigma wide range molecular weight marker (range 10–180 kDa).

### Brine shrimp bioassay: a hatchability test

Brine shrimp hatchability test was performed according to a method by Migliore et al. (1997). The brine shrimp (*Artemia salina*) eggs or cysts were hatched in sterile seawater (1 g cyst/l) at 28 °C, under conditions of continuous lighting and strong aeration. Different concentrations of marine actinobacteria extract (250, 500, 750, 1,000 μg/ml) were added along with the eggs. The number of free *nauplii* was calculated after each treatment.

## Results and discussion

### Screening of *Bacillus* VITRKHB for cellulolytic activity

A clear zone of hydrolysis (15.2 mm) observed on the CMC agar plate showed cellulolytic activity of this *Bacillus* VITRKHB. Several reports have described cellulase production from this particular genus isolated from terrestrial (Das et al. 2010; Aboul-Enein et al. 2010; Verma et al. 2012) as well as marine sources (Bhat and Bhat 1997; Mohapatra et al. 2003; Taylor et al. 2006).

### Effect of different carbon sources on cellulase production

**Fig. 1:**
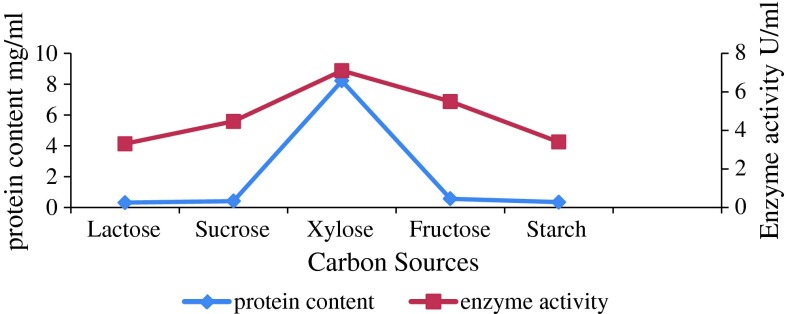
Effect of carbon sources on enzyme activity and protein content

Enzyme production and enzyme activity were found to be maximum when xylose was used as the carbon source (Fig. 1). Production was very low in the fermentation media containing starch. Carbon source and its pretreatment are the major cost-contributing factor for commercial enzyme production (Marsden and Gray 1986). A study on cellulase production by *Bacillus* sp. from terrestrial sources described cellulose as the best carbon source (Wei et al. 2011), while xylan proved to be the best carbon source for cellulase production from *Bacillus cereus* MRK1 (Mukesh Kumar et al. 2012). Sucrose gave the best result as compared to others during cellulase production from *Trichoderma viride* (Gautam et al. 2010). Lactose was used as the carbon source during mesophilic alkaline cellulase production from marine bacterium *Marinobacter* sp. MSI032 (Shanmughapriya et al. 2010). These studies suggest that cellulose is the significant carbon source for cellulase production from *Bacillus* sp. isolated from terrestrial sources. However, our study confirms that disaccharides and monosaccharides such as sucrose and xylose are better carbon supplements for cellulase production from marine source.

### Effect of different nitrogen sources on cellulase production

**Fig. 2:**
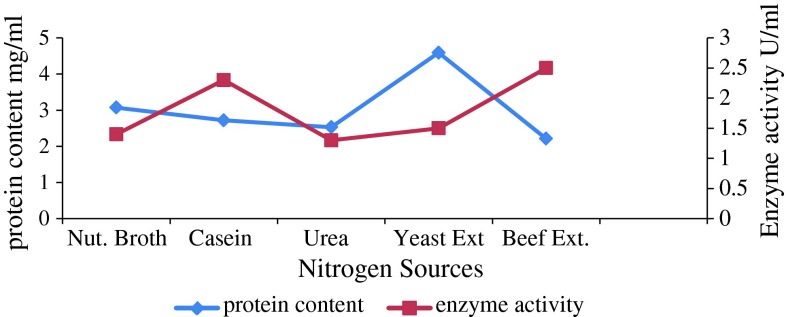
Effect of nitrogen sources on enzyme activity and protein content

Cellulase yield was maximum with yeast extract used as nitrogen source, although activity was maximum when beef extract was used (Fig. 2). This is in accordance with a report on cellulase production by *B. cereus* (Mukesh Kumar et al. 2012) that suggested yeast extract to be the best nitrogen source for cellulase production. However, a report also showed peptone as the best nitrogen source for cellulase production (Marsden and Gray 1986). Likewise, another report suggested yeast extract as nitrogen source for cellulase production from *T. viride* (Gautam et al. 2010). Peptone and casein were also reported as nitrogen sources for cellulase production from sponge-associated marine bacterium *Marinobacter* sp. MSI032 (Shanmughapriya et al. 2010). These studies suggest that yeast extract and peptone may be preferred for better cellulase production. However, our study suggests beef extract to be the appropriate nitrogen source for desirable enzyme activity.

### Optimization using response surface methodology (RSM)

Investigation of the mutual interactive effect of the independent variables, viz., xylose (A), beef extract (B), pH (C), NaCl (D), and temperature (E), on the production of cellulase from *Bacillus* VITRKHB was carried out and the optimal level of each variable was attained. An experiment with 31 runs was designed and performed with an incubation period of 24 h. ANOVA was carried out using a response surface quadratic model that gave the following equation:

$$\begin{aligned} {\text{R1}} & = + 1. 6 + 0.0 1 1\times {\text{A}} + 1. 7 5 1 {\text{E}} - 00 3\times {\text{ B }} - 1.0 2 3 {\text{E}} - 00 4\times {\text{C}} + 1. 1 6 7 {\text{E}} - 00 3\times {\text{D}} + 0.0 1 4\times {\text{E }} - 2.00 2 {\text{E}} - 00 3\times {\text{A}} \times {\text{B}} \\ + 8. 40 9 {\text{E}} - 00 3\times {\text{A}} \times {\text{C}} + 7. 3 3 8 {\text{E}} - 00 4\times {\text{A}} \times {\text{D }} + 3. 5 6 2 {\text{E}} - 00 3\times {\text{A}} \times {\text{E}} - 0.0 1 3\times {\text{B}} \times {\text{C}} + 7. 10 7 {\text{E}} - 3\times {\text{B}} \times {\text{D}} \\ + 0.0 10 \times {\text{B}} \times {\text{E}} - 4. 3 6 3 {\text{E}} - 00 3\times {\text{C}} \times {\text{D}} - 9. 50 7 {\text{E}} - 00 4\times {\text{C}} \times {\text{E}} + 0.0 20 \times {\text{D}} \times {\text{E}} \\ \end{aligned}$$

**Fig. 3:**
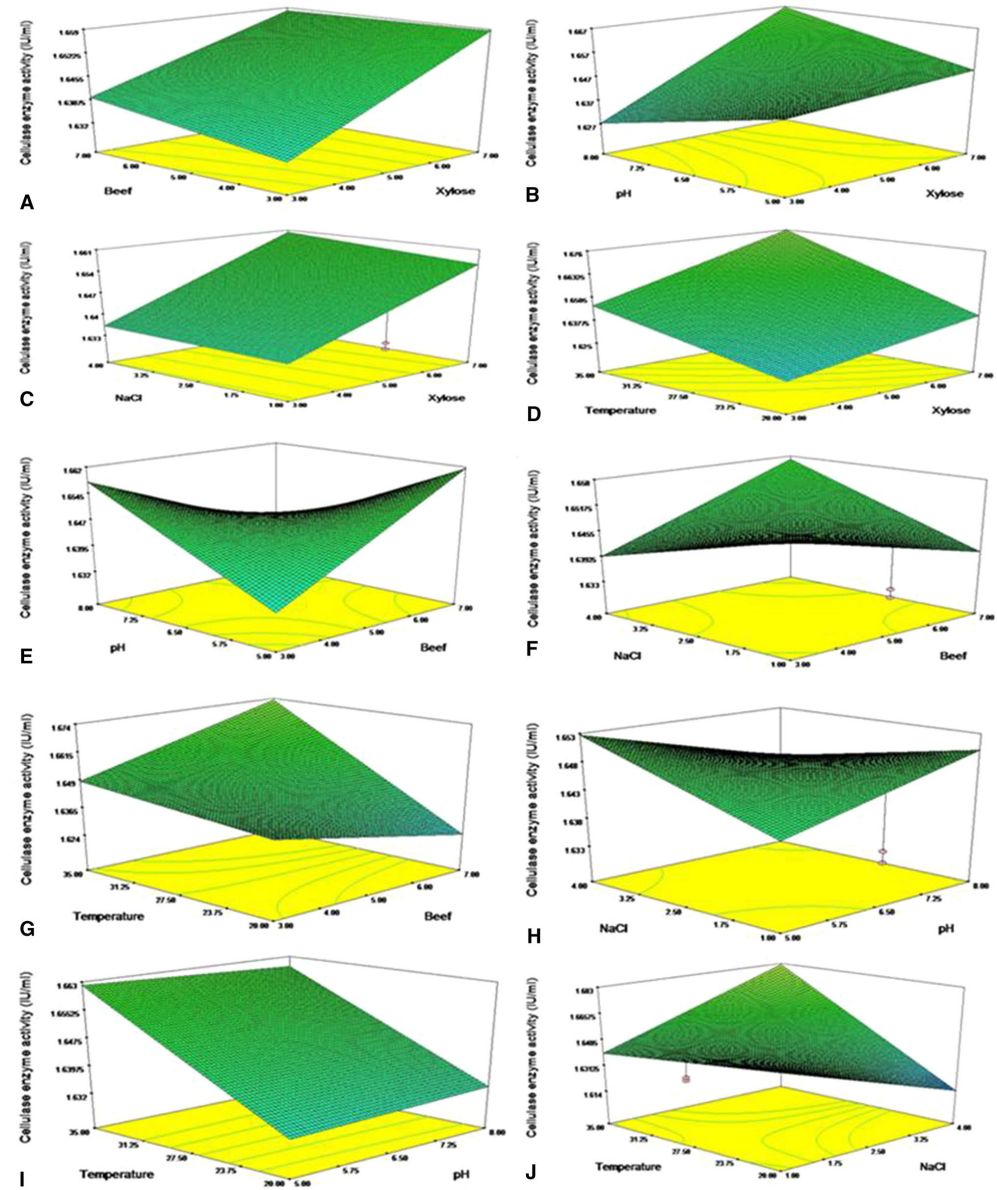
Effects of **a** beef extract and xylose, **b** pH and xylose, **c** NaCl and xylose, **d** temperature and xylose, **e** pH and beef extract, **f** NaCl and beef extract, **g** temperature and beef extract, **h** NaCl and pH, **i** temperature and pH, **j** temperature and NaCl

The outcome of ANOVA analysis was recorded. The *F* value was 1.44 and there was a 24.35 % chance that a “model *F* value” this large could occur due to noise. Values of “prob > *F*” <0.0500 indicated that the model terms were significant. DE was a significant model term of this analysis. Values >0.1000 indicate that the model terms are not significant. The “lack of fit *F* value” of 2.43 implied that lack of fit was not significant relative to the pure error. There was a 16.97 % chance that a “lack of fit *F* value” this large could occur due to noise. Non-significant lack of fit was good. In this study, all the linear, interactive effects of AB, AC, AD, AE, DE, BC, BD, BE, CD, CE, and DE were good for enzyme activity. The coefficient of determination (*R*2) for enzyme activity was calculated as 0.5905. The interactive effects of independent variables on enzyme were studied by plotting 3D surface curves. The 3D curves of the calculated enzyme activity for the interactions between the variables are shown in Fig. 3. The optimal levels of the significant variables for the maximum amylase production were: xylose 5 %, beef extract 6.90 %, pH 7.83, NaCl 1.17 %, and temperature 25.84 °C.

Reduction in the production cost of cellulase by optimization of growth conditions and the process is one of the major targets of cellulase research (Sukumaran et al. 2005) Optimization of fermentation parameters for the development of economically feasible bioprocesses has been a challenge (Hao et al. 2006). Effective experimental designs may lead to the development of the optimum process. The experimental designs developed by the combination of statistics and mathematics have played a significant role in the field of industrial biotechnology. Response surface methodology is one such method that employs statistical techniques for designing experiments, building models, evaluating combined interactive effect of different independent variables, and determining the optimum conditions. Performing statistically designed experiments, filling experimentally determined response data into a quadratic model, predicting response, and checking the significance of the model are the major steps of this process (Vohra and Satyanarayana 2002). RSM eases the process of optimization as it involves rapid screening of the optimal condition with lesser consumption of material and effort. Satisfactory optimization of microbial enzyme production becomes possible with the implication of the 3D plots for response surface which allows direct visualization of parameter interaction (Mullai et al. 2010). Earlier, RSM-mediated optimization was applied for the evaluation of the combined interactive effect of wheat bran, Avicel, soybean cake flour, and corn steep flour in the production of cellulase from mutant strain *T. reesei* WX-112. An increase in cellulase activity was observed (Mullai et al. 2010). Central composite design-based RSM was also used for optimizing the level of lignocelluloses and lactose for maximum cellulase production from agricultural waste (Muthuvelayudham and Viruthagiri 2010). Another study reports optimization of media composition (palm oil mill effluent as basal media) from *T. reesei* RUT-C30 using face-centered central composite design under RSM (Rashid et al. 2009). According to these reports, RSM-mediated optimization of cellulase production results in enhanced enzyme yield and activity as compared to the classical method of optimization. Hence, in the present study, we endeavored to apply RSM to examine the effect of combined interaction of different variables, starch, yeast extract, pH, NaCl, and temperature on the production of cellulase. This technique was found to be efficient for the rapid optimization of the process parameters for cellulase production in our study. After optimization, production of cellulase from marine *Bacillus* VITRKHB was found to be economical, with an increased enzyme yield and activity.

### Partial purification of enzyme

RSM-optimized medium was further employed for the production of cellulase. Thereafter, the crude extract was subjected to purification steps. Total protein, enzyme activity, specific activity, and fold purification were calculated (Table 2). The enzyme was purified to 1.30-fold with specific activity of 1.60 U/mg after ammonium sulfate precipitation. It increased to 1.60 U/mg after dialysis with a fold purification of ~1.55. The dialyzed enzyme was subjected to DEAE ion exchange chromatography. The fold purification increased to 1.90 and specific activity was found to be 1.962. The enzyme yield after ion exchange chromatography was 41.3 %, which was quite high. 1.6-fold purification was attained after ion exchange chromatography.

**Table 2:** Purification of crude extract for cellulase isolated from *Bacillus* VITRKHB

Purification steps	Total protein (mg/ml)	Enzyme activity (U/ml)	Specific activity (U/mg)	Fold purification	Yield (%)
Crude extract	3.10	3.80	1.225	1	100
Ammonium sulfate precipitation	2.14	3.43	1.60	1.30	90.2
Dialysis	1.60	3.05	1.90	1.55	80.2
Ion exchange	0.80	1.57	1.962	1.60	41.3

**Fig. 4:**
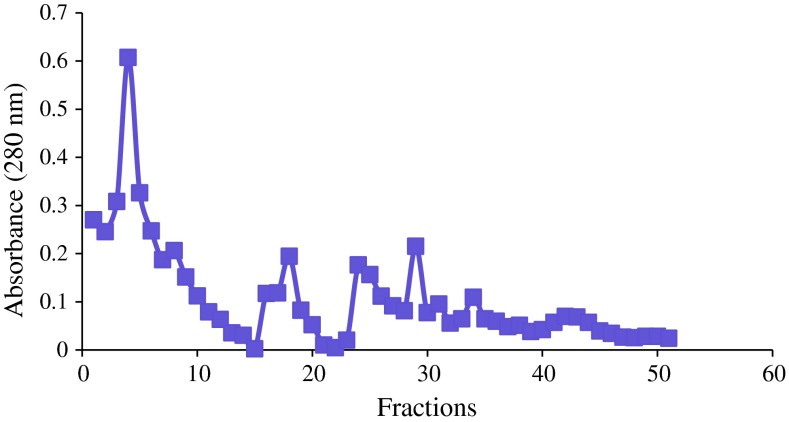
Purification of cellulase using DEAE cellulose column

Trivedi et al. (2011) reported a yield of 25.03 % with a purification of 22.31-fold during alkali halotolerant cellulase production from marine bacterium *B. flexus* isolated from *Ulva lactuca*. Likewise, a study showed 25.10 % recovery by using DEAE cellulose and Sephadex G-200 columns for purification of themoactive cellulase from thermophilic *Actinomycetes* (Verma et al. 2012). These previously published reports indicate a minimum loss of enzyme after each purification step and the effectiveness of the purification method applied in this study. The elution pattern of the enzyme and a broad active peak were achieved when the dialyzed sample was passed through a DEAE cellulose column with 0.1 M Tris–HCI buffer with Nacl (pH 7.83). The fourth peak contained the highest protein concentration (0.80 mg/ml) (Fig. 4).

### SDS-PAGE

**Fig. 5:**
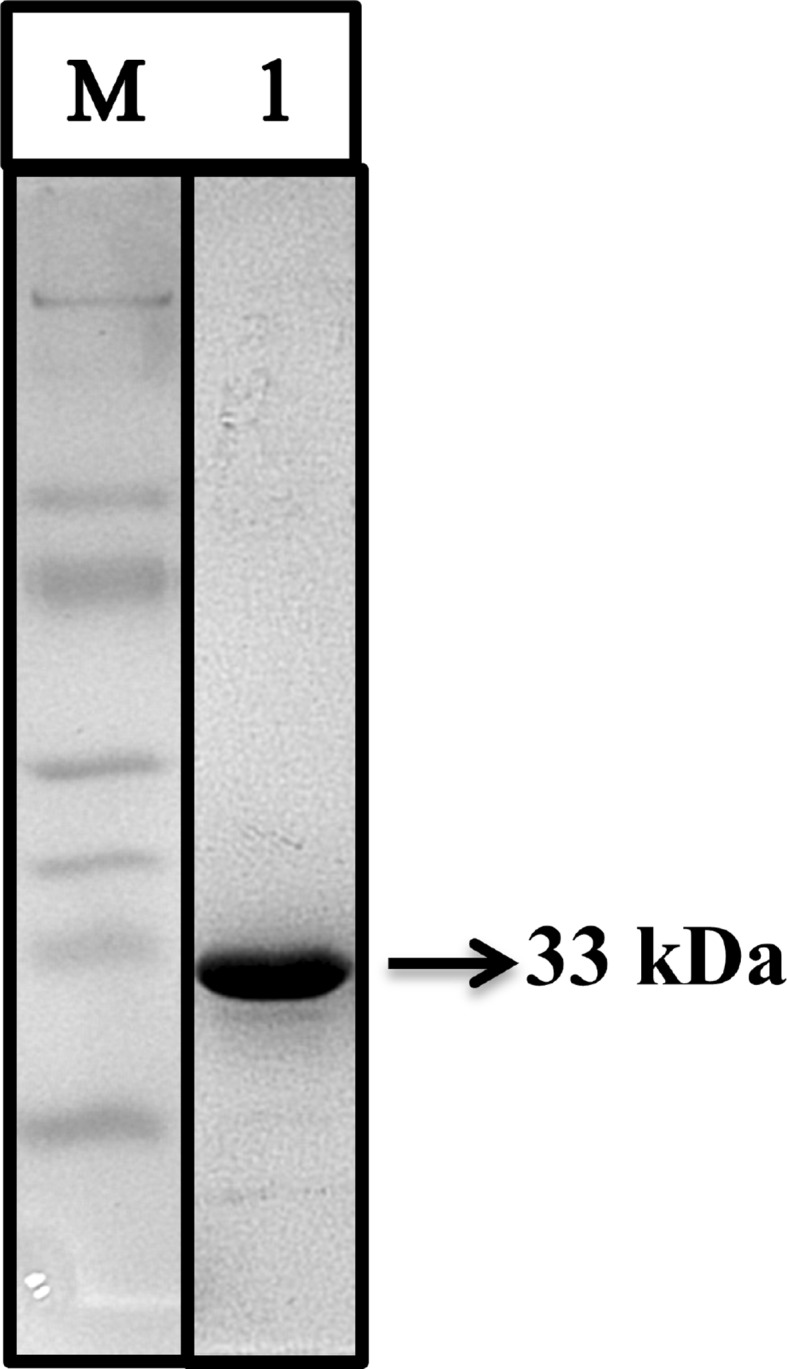
SDS-PAGE of purified cellulase from *Bacillus* VITRKHB

The molecular weight of purified protein was found to be 33 kDa by SDS-PAGE (Fig. 5). The molecular weight was similar to that of some of the other low molecular weight (25–45 kDa) endoglucanases produced by *Bacillus* spp. (Mawadza et al. 2000; Yin Li et al. 2010; Saha 2004; Lucas et al. 2001; Aa et al. 1994).

### Brine shrimp hatchability test

*Artemia* embryos were exposed to isolated marine eubacterial cellulase and the percentage of toxicity was checked at 12 and 24 h of exposure. There was no significant change in the hatchability of *Artemia* embryos observed up to 1,000 μg. *Artemia* embryos are highly sensitive to toxins at the early developmental stages. The cellulase extract isolated from marine *Bacillus* VITRKHB did not show any toxicity when subjected to this preliminary toxicity test. Hence, it can be considered as safe for use as food additives in animal feed and also in textiles and detergents.

## Conclusion

Cellulase finds a wide range of application in research and development and in the industrial sector. It is mainly employed in animal feed, textiles, detergent industry, and fuel production from biomass. With increase in demand, its higher yield, novel activity, and economical production are desirable. This study reports optimized production and purification of cellulase from marine *Bacillus* VITRKHB. Cellulase was produced and partially purified stepwise by a combination of ammonium sulfate precipitation, dialysis, and DEAE ion exchange chromatography to 1.6-fold and its specific activity was found to be 1.92 IU/mg. The molecular weight of purified protein was found to be 33 kDa by SDS-PAGE. The highest enzyme activity was observed in the media containing xylose as carbon source and beef extract as nitrogen source during this study. Response surface methodology-mediated media optimization was carried out for visualizing the combined interactive effects of different variables, i.e., xylose 5.0 %, beef extract 6.9 %, pH 7.83, NaCl 1.17, and temperature 25.84 °C on enzyme activity at 24 h of incubation. The brine shrimp hatchability test indicated non-toxicity of our enzyme. Hence, the strain *Bacillus* VITRKHB used in this study is potent for cellulase production and the parameters optimized are suitable for the economic production of this enzyme. In addition to this, the enzyme isolated during this study displays good industrial efficacy.

## References

[CR1] Aa K, Flengsrud R, Lindahl V, Tronsmo A (1994). Characterisation of production and enzyme properties
of an endo-β-1,4-glucanase from *Bacillus
subtilis* CK-2 isolated from compost soil. Antonie Van Leeuwenhoek.

[CR2] Aboul-Enein A, Abou elalla F, Serour E, Hussien T (2010). Purification and characterization of a novel
thermoactive cellulase from thermophilic Actinomycetes isolated from soil sample
of Egypt. Int J Acad Res.

[CR15] Ashwini K, Kumar G, Loganathan L, Bhaskara Rao KV (2011). Optimization, production and partial purification of
extracellular α-amylase from *Bacillus* sp.
*Marini*. Arch Appl Sci Res.

[CR3] Aygan A, Arikan B (2008). A new halo-alkaliphilic, thermostable endoglucanase
from moderately halophilic *Bacillus* sp. C14
isolated from Van soda lake. Int J Agric Biol.

[CR4] Bhat M, Bhat S (1997). Cellulase degrading enzymes and their potential
industrial applications. Biotechnol Adv.

[CR5] Buchert J, Surnakki A, Tenkanen M, Viikari L (1996) Enzymatic characterization of pulp. In: Jeffries TW, Viikari L (eds) Enzymes for pulp and paper processing ACS Symp Ser, vol 655, p 38–48

[CR6] Chakraborty S, Khopadea A, Kokarea CK, Mahadika KS (2009). Isolation and characterization of novel-amylase from
marine *Streptomyces* sp. D1. J Mol Catal B: Enzymatic..

[CR7] Das A, Bhattacharya S, Murali L (2010). Production of cellulase from Thermophilic *Bacillus* sp. isolated from cow dung. AM Eurasian J Agric Environ Sci.

[CR8] Denison DA, Koehn RD (1977). Cellulase activity of *Poronia
Oedipus*. Mycologia.

[CR9] Eichler J (2001). Biotechnological uses of archaeal
extremozymes. Biotechnol Adv.

[CR10] Elibol M (2003). Optimization of medium composition for actinorhodin
production by *Streptomyces coelicolor* A3 (2)
with response surface methodology. Process Biochem.

[CR11] Gautam SP, Bundela PS, Pandey AK, Jamaluddin, Awasthi MK, Sarsaiya S (2010). Optimization of the medium for the production of
cellulase by the *Trichoderma viride* using
submerged fermentation. Int J Environ Sc.

[CR12] Gupta MN, Roy I (2002). Applied biocatalysis: an overview. Indian J Biochem Biophys.

[CR13] Hao XC, Yu XB, Yan ZL (2006). Optimization of the medium for the production of
cellulase by the mutant *Trichoderma reesei*
WX-112 using response surface methodology. Food Technol Biotechnol.

[CR14] Herbert RA (1992). A perspective on the biotechnological potential of
extremophiles. Trends Botechnol.

[CR16] Laemmli UK (1970). Cleavage of structural proteins during the assembly of
the head of bacteriophage T4. Nature.

[CR17] Liu BL, Tzeng YM (1998). Optimization of growth medium for production of spores
from *Bacillus thuringiensis* using response
surface methodology. Bioprocess Eng.

[CR18] Lowery OH, Rosebrough NJ, Farr AL, Randall RJ (1951). Protein measurement by Folin phenol
reagent. J Biol Chem.

[CR19] Lucas R, Robles A, Garcia MT, Alvarez De Cienfuegos G, Galvez A (2001). Production, purification, and properties of an
endoglucanase produced by the hyphomycete Chalara (syn. *Thielaviopsis*) *paradoxa*
CH32. J Agric Food Chem.

[CR20] Marhuenda-Egea FC, Bonete MJ (2002). Extreme halophilic enzymes in organic
solvents. Curr Opin Biotechnol.

[CR21] Marsden WL, Gray PP (1986). Enzymatic hydrolysis of cellulose in lignocellulosic
material. CRC Crit Rev Biotechnol.

[CR22] Mawadza C, Hatti-Kaul R, Zvauya R, Mattiasson B (2000). Purification and characterization of cellulases
produced by two *Bacillus*
strains. J Biotechnol.

[CR23] Migliore L, Civitareale C, Brambilla G, Di delupis GD (1997). Toxicity of several important agricultural antibiotics
to Artemia. Water Res.

[CR24] Mohapatra BR, Bapuji M, Sree A (2003). Production of industrial enzymes amylase,
carboxymethylcellulase and protease by bacteria isolated from marine sedentary
organisms. Acta Biotechnol.

[CR25] Mukesh Kumar DJ, Poovai PD, Puneeth Kumar CL, Sushma Saroja Y, Manimaran A, Kalaichelvan PT (2012). Optimization of *Bacillus
cereus* MRK1 cellulase production and its Biostoning
activity. Der Pharm Lett.

[CR26] Mukhopadhyay A, Redding AM, Rutherford BJ, Keasling JD (2008). Importance of systems biology in engineering microbes
for biofuel production. Curr Opin Biotechnol.

[CR27] Mullai P, Fathima NSA, Rene ER (2010). Statistical analysis of main and interaction effects
to optimize xylanase production under submerged cultivation
conditions. J Agric Sci.

[CR28] Muthuvelayudham R, Viruthagiri T (2010). Application of central composite design based response
surface methodology in parameter optimization and on cellulase production sing
agricultural waste. Int J Chem Biol Eng.

[CR29] Rashid SS, Alam MZ, Karim MIA, Salleh MH (2009). Optimization of the nutrient supplements for cellulase
production with the basal medium palm oil mill effluent. World Acad Sci Eng Tech.

[CR30] Richa K, Bose H, Singh K, Karthik L, Kumar G, Bhaskara Rao KV (2013). Response surface optimization for the production of
marine eubacterial protease and its application. Res J Biotechnol.

[CR31] Saha BC (2004). Production, purification and properties of
endoglucanase from a newly isolated strain of *Mucor
circinelloides*. Process Biochem.

[CR32] Shanmughapriya S, Kiran GS, Selvin J, Thomas TA, Rani C (2010). Optimization, purification, and characterization of
extracellular mesophilic alkaline cellulase from sponge-associated *Marinobacter* sp. MSI032. Appl Biochem Biotechnol.

[CR33] Sukumaran KR, Singhania RR, Pandey A (2005). Microbial cellulases production, applications and
challenge. J Sci Ind Res.

[CR34] Taylor LE, Henrissat B, Coutinho PM, Ekborg NA, Hutcheson SW, Weiner RM (2006). Complete cellulase system in the marine bacterium
*Saccharophagus degradans* strain
2-40^T^. J Bacteriol.

[CR35] Trivedi N, Gupta V, Kumar M, Kumari P, Reddy CRK (2011). An alkali-halotolerant cellulase from *Bacillus flexus* isolated from green seaweed *Ulva lactuca*. Carbohyd Polym.

[CR36] Verma V, Verma A, Kushwaha A (2012). Isolation and production of cellulase enzyme from
bacteria isolated from agricultural fields in district Hardoi, Uttar Pradesh,
India. Adv Appl Sci Res.

[CR37] Vohra A, Satyanarayana T (2002). Statistical optimization of the medium components by
response surface methodology to enhance phytase production by *Pichia anomala*. Process Biochem.

[CR38] Wang CY, Hsieh YR, Ng CC, Chan H, Lin HT, Tzeng WS, Shyu YT (2009). Purification and characterization of a novel
halostable cellulase from *Salinivibrio* sp.
strain NTU-05. Enzyme Microb Technol.

[CR39] Wei ZJ, Zhou LC, Chen H, Chen GH (2011) Optimization of the fermentation conditions for 1-deoxynojirimycin production by *Streptomyces lavendulae* applying the response surface methodology. Int J Food Eng 7:1–10

[CR40] Yin Li J, Lin HH, Xiao ZR (2010) Purification and characterization of a cellulase from *Bacillus subtilis* YJ1. J Mar Sci Technol 18:466–471

[CR41] Zambare V (2011). Optimization of amylase production from *Bacillus* sp. using statistics based experimental
design. Emir J Food Agric.

